# ASIC1a stimulates the resistance of human hepatocellular carcinoma by promoting EMT via the AKT/GSK3β/Snail pathway driven by TGFβ/Smad signals

**DOI:** 10.1111/jcmm.17288

**Published:** 2022-04-14

**Authors:** Yinci Zhang, Niandie Cao, Jiafeng Gao, Jiaojiao Liang, Yong Liang, Yinghai Xie, Shuping Zhou, Xiaolong Tang

**Affiliations:** ^1^ Medcial School Anhui University of Science & Technology Huainan China; ^2^ Institute of Environment‐friendly Materials and Occupational Health of Anhui University of Science and Technology Wuhu China; ^3^ Huai’an Hospital Affiliated of Xuzhou Medical College and Huai’an Second Hospital Huai’an China; ^4^ First Affiliated Hospital Anhui University of Science & Technology Huainan China

**Keywords:** acid‐sensing ion channel 1a, AKT/GSK‐3β/Snail pathway, chemosensitivity, epithelial to mesenchymal transition, hepatocellular carcinoma, invasion, migration, multidrug resistance

## Abstract

Multidrug resistance is the main obstacle to curing hepatocellular carcinoma (HCC). Acid‐sensing ion channel 1a (ASIC1a) has critical roles in all stages of cancer progression, especially invasion and metastasis, and in resistance to therapy. Epithelial to mesenchymal transition (EMT) transforms epithelial cells into mesenchymal cells after being stimulated by extracellular factors and is closely related to tumour infiltration and resistance. We used Western blotting, immunofluorescence, qRT‐PCR, immunohistochemical staining, MTT, colony formation and scratch healing assay to determine ASIC1a levels and its relationship to cell proliferation, migration and invasion. ASIC1a is overexpressed in HCC tissues, and the amount increased in resistant HCC cells. EMT occurred more frequently in drug‐resistant cells than in parental cells. Inactivation of ASIC1a inhibited cell migration and invasion and increased the chemosensitivity of cells through EMT. Overexpression of ASIC1a upregulated EMT and increased the cells’ proliferation, migration and invasion and induced drug resistance; knocking down ASIC1a with shRNA had the opposite effects. ASIC1a increased cell migration and invasion through EMT by regulating α and β‐catenin, vimentin and fibronectin expression via the AKT/GSK‐3β/Snail pathway driven by TGFβ/Smad signals. ASIC1a mediates drug resistance of HCC through EMT via the AKT/GSK‐3β/Snail pathway.

## 1 INTRODUCTION

Hepatocellular carcinoma (HCC) is the sixth commonest widespread cancer^1^, ^2^ and is a fatal malignant tumour with a high recurrence rate and chemoresistance.^3^ Chemotherapy is a primary treatment for HCC, but drug resistance severely reduces its effectiveness.^4^ Many molecular mechanisms underlying the resistance of chemotherapeutic treatments of HCC have been proposed, but the problem persists. Therefore, understanding the mechanism of chemotherapy resistance is key to improved treatment of HCC.

The induction and maintenance of an abnormal extracellular acidic microenvironment is a key in the formation and progression of malignant tumours.^5^ The acidic microenvironment is mainly caused by glycolysis and hypoxia of tumour cells. Glycolysis produces large amounts of acidic products, thus forming an acidic environment that is toxic to adjacent normal tissue cells. The hypoxia caused by the growth of tumour cells can activate hypoxia‐inducible factor 1 (HIF‐1) to make tumour cells adapt to the acid environment outside the tumour, which in turn facilitates invasion of tumour tissues into adjacent normal tissues.^6^, ^7^ Therefore, the external acidic environment of tumour tissue accelerates tumour metastasis and malignant development, whereas tumour metastasis is the initiating factor in tumour resistance.^8^ The extracellular acidic environment can reduce the apoptotic potential, change the genetic properties and increase the activity of multidrug transporters to make tumour cells resistant to the effects of chemotherapy drugs.^9^, ^10^ The acidic extracellular environment also can increase the expression of interleukin 8 (IL‐8), vascular endothelial growth factor, cathepsin B, matrix metalloproteinase 2 (MMP2) and matrix metalloproteinase 9 (MMP9).^11^, ^12^ In short, the low‐acid extracellular environment contributes to chemotherapy resistance, but the molecular mechanism is unknown.

Acid‐sensitive ion channels (ASICs) are present in various neurons and non‐neuronal tissues. ASICs have six subunits, 1a, 1b, 2a, 2b, 3 and 4, and can mediate the influx of Ca^2+^.^13^, ^14^ As proton‐gated channels, ASICs are related to many pathophysiological conditions that are regulated by pH.^15^, ^16^ thus indicating that ASICs have important physiological and pathophysiological activities.^15^, ^16^ Due to high glucose metabolism and poor perfusion, the tumour microenvironment has low acidity.^17^ Therefore, ASIC1a, as the most conserved acid‐sensitive ion channel molecule, is related to cancer and to the proliferation and migration of various cells.^18^, ^19^ ASIC1a also stimulates the drug resistance of HCC via the Ca^2+^/PI3K/AKT pathway.^20^ However, the ways in which ASIC1a participates in HCC resistance and the downstream signalling pathway of AKT have not been studied in depth.

Epithelial–mesenchymal transition (EMT), which is induced by the tumour microenvironment, is one of the most important mechanisms for initiating and promoting tumour cell metastasis, and it is important in chemotherapy resistance. The acidic microenvironment can promote the EMT of lung cancer cells and melanoma cells to promote tumour progression and metastasis.^21^, ^22^

The AKT pathway can mediate tumour cells to escape apoptosis, thereby inducing drug resistance.^23^ Glycogen synthase kinase 3 beta (GSK3β) is a multifaceted kinase that is a key regulator of numerous cellular processes and participates in multiple pathways.^24^, ^25^ GSK3β) has been studied extensively because of its role in the Wnt signalling pathway‐related EMT. The AKT/GSK‐3β/Snail pathway can induce EMT progression of tumour cells,^26^, ^27^ of which the activation of Akt promotes the phosphorylation of GSK‐3β, suppressing the expression of Snail, which ultimately facilitates EMT.^28^, ^29^ In HCC cells, whether EMT is involved in drug resistance via AKT/GSK‐3β/Snail pathway is unknown.

In this research, we examined the degree of activation of ASIC1a in the extracellular low‐acid microenvironment and its role in tumour resistance. We explored whether the acidic microenvironment activates EMT and further participates in drug resistance in HCC.

## 2 MATERIALS AND METHODS

### 2.1 Collection of patient tissue

This study was approved by the Human Research Ethics Committee of the First Affiliated Hospital of Anhui University of Science and Technology (China) and was conducted in accordance with the Declaration of Helsinki. Informed consent was obtained from each subject.

### 2.2 Cell source and culture

The HepG2 (H) and Bel7402 (B) cell lines were obtained from Mingjin Biotechnology Co., Ltd. (Shanghai, China) and certified with the short tandem repeat (STR) method. The oxaliplatin (OXA)‐resistant HepG2 cell line (H^R^) that can stably grow in 20 μM oxaliplatin and the 5‐fluorouracil (5FU)‐resistant Bel7402 cell line (B^R^) that can stably grow in 2 mM 5‐fluorouracil were induced by the concentration gradient method. Cells were cultured in RPMI‐1640 containing 10% foetal bovine serum (Sijiqing Bioofengineering Materials, Hangzhou, China) and incubated in 5% CO_2_ at 37°C. H^R^ and B^R^ cells were cultured in pH 6.5 and pH 7.4.

### 2.3 Reagents and chemicals

PcTx1 (a potent and selective ASIC1α blocker, Psalmotoxin 1) and GN25 (a novel inhibitor of Snail‐p53 binding) were procured from ApexBio Technology (Houston, USA). MK2206 (a specific inhibitor of AKT), TWS119 (a specific inhibitor of GSK3β) and Galunisertib (LY2157299) (TGF‐β/Smad inhibitor) were obtained from Selleck Chemicals (Houston, TX, USA). C19 (an inhibitor of EMT) was obtained from Shanghai Dongcang Biological Technology Co., Ltd. (Shanghai, China). OXA and 5FU were obtained from Sigma‐Aldrich (St. Louis, USA). These reagents and chemicals were dissolved in dimethyl sulfoxide at −80℃ until use and then diluted to a suitable working concentration with RPMI‐1640 medium. TGF‐β1 was obtained from PeproTech (Rocky Hill, NJ). TRIzol reagent was bought from Invitrogen Corp. (Carlsbad, CA, USA). First Strand cDNA Synthesis Kit was acquired from Thermo Fisher Scientific (Waltham, MA, USA).

### 2.4 Western blotting assay

Cells were collected and lysed by strong RIPA lysate containing protease inhibitors (1: 50 dilution, Bioss Biotechnology Company, Beijing, China). BCA‐200 was used to determine the concentration of protein in the lysate. The membranes were sealed with 5% skim milk for 1 h then incubated with anti‐ASIC1a (1: 200; Alomone Laboratories, Jerusalem, Israel), anti‐AKT, Phospho‐AKT, GSK3β, Phospho‐GSK3β, Snail, Smad2/3, Phospho‐Smad2/3, TGF‐β1, β‐actin (1:1000, Cell Signalling Technology, Danvers, MA, USA), anti‐MMP2, MMP9, α‐catenin, β‐catenin, E‐cadherin, vimentin and fibronectin (1:500, Abcam, Cambridge, UK) antibodies overnight at 4°C. The membranes were incubated in TBST buffer solution containing the secondary antibody (1:4000) at room temperature for 1 h. The results displayed by the gel imaging system were compared with the percentage of the control signal to correct for the difference between the imprints.

### 2.5 Quantitative real‐time PCR analysis

Total RNA was collected from HCC cells and tissues and used to synthesize cDNA as manufacturer's protocol. The primers applied for qRT‐PCR were as follows:

ASIC1a expression were 5′‐ATGGAAAGTGCTACACGTTCAA‐3′(forward),

5′‐GTTCATCCTGACTATGGATCTGC‐3′(reverse);

GAPDH expression were 5′‐GGAGCGAGATCCCTCCAA AAT‐3′(forward),

5′‐GGCTGTTGTCATACTTCTCATGG‐3′(reverse).

The PCR was performed under the conditions of 95°C for 10 min, 40 cycles at 95°C for 15 s, 60°C for 30 s and 72°C for 30 s.

### 2.6 Cell morphology analysis

The cells at a density of 5 × 10^4^/well were inoculated on a 24‐well plate. After 24 h, representative images were taken at ×100 magnification. Finally, the local area of images was enlarged to show the morphology of the cells more clearly.

### 2.7 Immunofluorescence staining

The anti‐ASIC1a (1:200) antibody was applied according to our described method.^30^ Alexa Fluor 488‐conjugated goat anti‐rabbit IgG (H+L) (1: 1000; Abcam, Cambridge, UK) was used as fluorescent secondary antibody. For nuclear staining, cells were incubated with DAPI for 10 min.

### 2.8 Immunohistochemistry staining

Cells at a density of 3 × 10^5^/ml were inoculated on 24‐well plate‐containing slides. Endogenous peroxidase was inactivated with 3% H_2_O_2_ at 37°C for 10 min. The cells were fixed with absolute ethanol and blocked with 5% bovine serum albumen at 37°C for 10 min. Anti‐ASIC1a (1:200) was added and incubated overnight at 4°C. Biotin‐labelled secondary antibody was added dropwise and incubated at 37°C for 30 min. Horseradish peroxidase‐labelled streptomycin avidin working solution was dripped and incubated at 37°C for 30 min. Colour was developed with diaminobenzidine, and the slides were washed thoroughly with tap water and counterstained with haematoxylin.

### 2.9 MTT and colony formation assay

After drug treatment of each group for various times and concentrations, cell viability was measured with MTT and colony formation assay according to our described method.^30^ Cell cloning ability is expressed as a percentage of total cells forming a clonal mass.

### 2.10 Scratch healing assay

The cells at a density of 1.2 × 10^6^/ml were inoculated on a 12‐well plate. A gap between cells was created by scraping the bottom of each well with a 20‐μl sterile pipette tip. At intervals of 0 and 48 h, gaps between cells in the wells were quantitatively analysed with ImageJ Software to measure the scratch length.

### 2.11 Transwell invasion assay

BD matrigel (Solarbio, Shanghai) at 4°C was added to serum‐free medium at a ratio of 1:5 and mixed at 4°C on an ice bath. 100 μl of the mixed solution was added to the upper chamber and placed in a 37°C incubator for 5 h. The cell suspension was prepared in accordance with our described method.^31^

### 2.12 Silencing of ASIC1a

ASIC1a‐specific shRNA lentiviral particles (Genechem, China) were transfected into cells according to the manufacturer's protocol. The shRNA for gene silencing is

ASIC1a shRNA: CCGGCTATGGAAAGTGCTACACGTTC

TCGAGAACGTGTAGCACTTTCCATAGTTTTT

### 2.13 Overexpression of ASIC1a

ASIC1a gene‐lentiviral particles (Genechem, China) were transfected into cells according to the manufacturer's protocol. The primer for ASIC1a overexpression is

5′‐GAGGATCCCCGGGTACCGGTCGCCACCAT

GGAACTGAAGGCCGAGGAGGAG‐3′ (forward)

5′‐TCCTTGTAGTCCATACCGCAGGTAAAGTCCTCGAACGTG‐3′ (reverse)

### 2.14 Statistical analysis

All experiments were performed at least in triplicate and measured in three independent experiments. The data were expressed as mean ± SD. The means of two groups were compared by use of Student's t‐test, and three or more groups were compared by use of one‐way ANOVA. *p* < 0.05 was considered statistically significant. GraphPad Prism 5 was used for all analyses.

## 3 RESULTS

### 3.1 ASIC1a is overexpressed in HCC tissue and significantly upregulated in resistant cells

**FIGURE 1:**
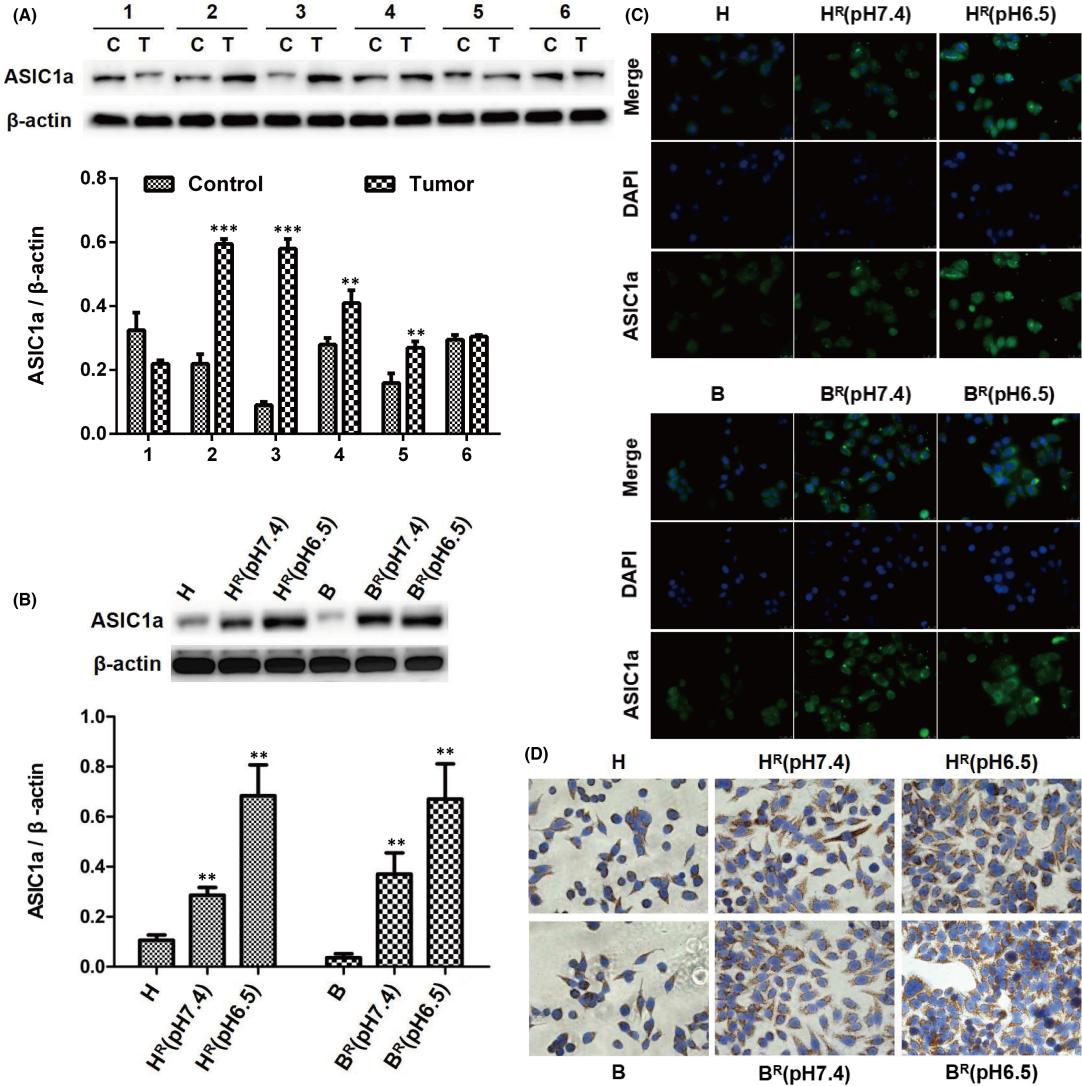
ASIC1a is overexpressed in HCC tissue and significantly upregulated in resistant cells. (A) Western blotting analysis of ASIC1a protein expression levels in HCC tissues and adjacent non‐tumour tissues. Data were expressed as the mean ± SD, *n* = 6. **p < 0.01 and ***p < 0.001 all versus Control group. C: Control (adjacent non‐tumour tissue), T: Tumour (HCC tissue). (B) Western blotting analysis of ASIC1a protein expression levels in HCC cells. Data were expressed as the mean ± SD, *n* = 3. **p < 0.01 all versus H group. (C) Expression levels and the cell distribution of ASIC1a in HCC cells were measured by immunofluorescence staining. Representative images were taken at ×400 magnification. (D) Expression levels and the cell distribution of ASIC1a in HCC cells were measured by immunohistochemistry staining. Representative images were taken at ×400 magnification

We determined the levels of ASIC1a in six pairs of HCC tissues and adjacent non‐tumour tissues by Western blotting and qRT‐PCR. As displayed in Figure 1A, ASIC1a was overexpressed in four pairs of tumour tissues, expressed at low levels in one pair of tumour tissues, and not significantly expressed in one pair of tumour tissues. The mRNA level of ASIC1a in HCC tissues was higher than in adjacent non‐tumour tissues (Figure S1A). This result is not significantly different from that in previous studies.^20^

We also found that the levels of ASIC1a were overexpressed in HCC‐resistant cells compared with levels in sensitive cells by Western blotting (Figure 1B) and qRT‐PCR (Figure S1B), suggesting that ASIC1a may be involved in tumour resistance. To further evaluate the level of ASIC1a in the drug‐resistant HCC cells H^R^ and B^R^, the expression of ASIC1a was determined with immunohistochemistry and immunofluorescence staining. The expression level of membrane ASIC1a was upregulated in H^R^ and B^R^ cells, especially at pH 6.5 (Figure 1C,D). A morphological cellular change (mesenchymal phenotype) in resistant HCC cells, especially at pH 6.5, also was found (Figure S2). These results not only indicate that ASIC1a is overexpressed in the drug‐resistant of HCC cells H^R^ and B^R^, but also reveal that a low pH in out of cell stimulates morphological cellular change (mesenchymal phenotype).

### 3.2 EMT is correlated with high expression of ASIC1a and drug resistance of HCC

**FIGURE 2:**
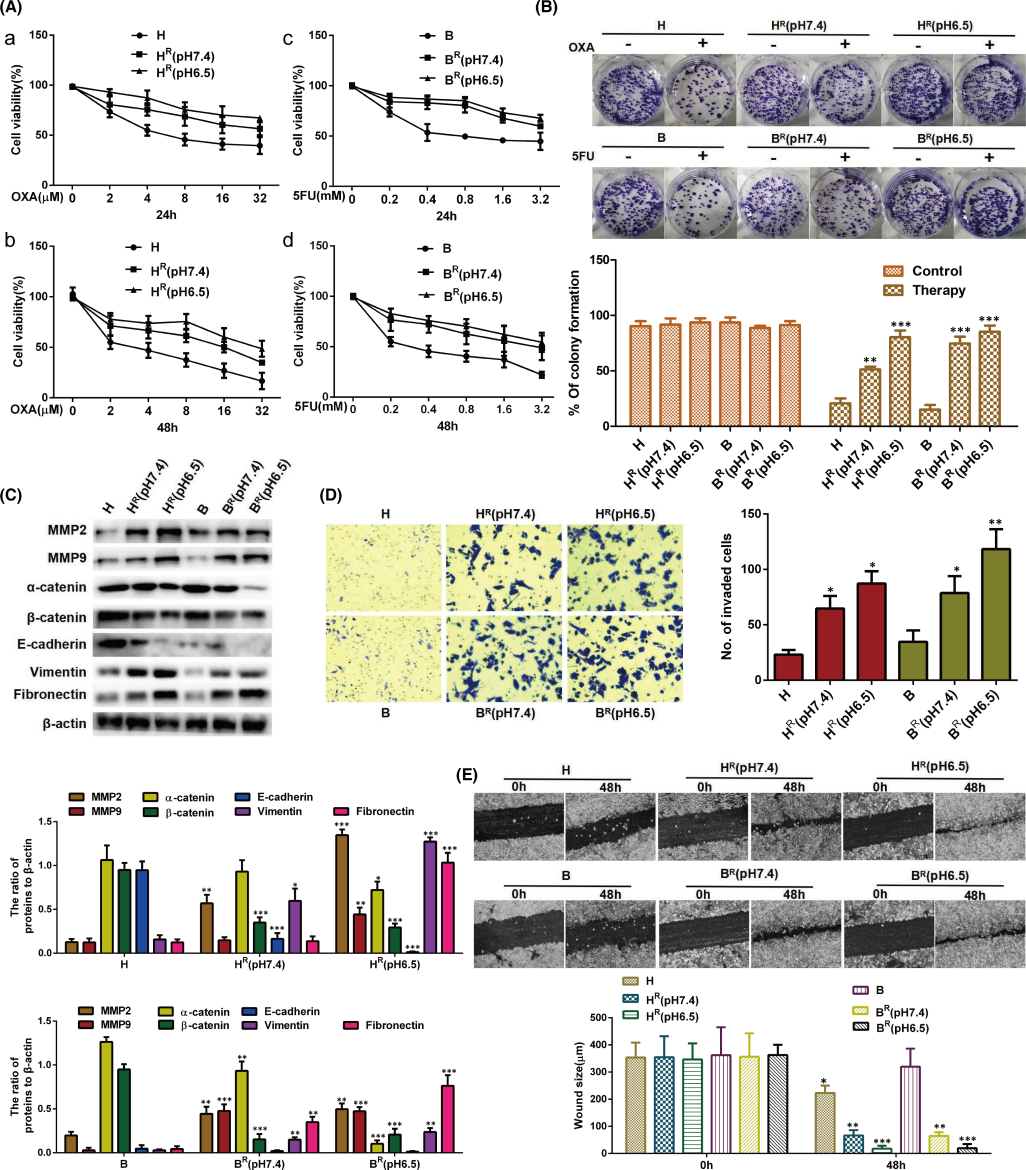
EMT is correlated with high expression of ASIC1a and drug resistance of HCC. (A) MTT analysis of the cell viability. Data were expressed as the mean ± SD, *n* = 3. (B) Representative images of the colony formation assay in HCC cells. The percentage of colony formation was calculated. Data are presented as mean ±SD, *n* = 3. **p < 0.01 and ***p < 0.001 all versus H or B therapy group. (C) Western blotting analysis of EMT marker molecule protein levels in HCC cells. Data were expressed as the mean ± SD, *n* = 3. *p < 0.05, **p < 0.01 and ***p < 0.001 all versus H group. (D) Invasion abilities of different group cells were determined by transwell invasion assays. Representative images were taken at ×200 magnification. Data were expressed as the mean ± SD, *n* = 3. *p < 0.05 and **p < 0.01 all versus H group. (E) Migration abilities of different group cells were examined by wound‐healing assays. Representative images were taken at ×100 magnification. Data were expressed as the mean ± SD, *n* = 3. *p < 0.05, **p < 0.01 and ***p < 0.01 all versus 0 h group

EMT has a crucial role in cancer progression, metastasis and drug resistance.^32^, ^33^ When ASIC1a is transferred to the cell membrane, it can promote the progression of cancer and induce drug resistance.^20^, ^34^ To further determine the role of EMT and the correlation between ASIC1a and EMT in tumour resistance, we cultured H^R^ and B^R^ cells in pH 6.5 and pH 7.4 medium. We determined the viability of the drug‐resistant and sensitive HCC cells by MTT. The results revealed that H^R^ cells have stronger cell viability than H cells after treatment with 2, 4, 8, 16 and 32 μM OXA for 24 h and 48 h, especially at pH 6.5 (Figure 2Aa,b). Similarly, B^R^ cells have stronger cell viability than B cells after treatment with 0.2, 0.4, 0.8, 1.6 and 3.2 mM 5‐FU for 24 h and 48 h, especially at pH 6.5 (Figure 2Ac,d). Next, the clonogenic ability of H^R^ cells cultured in pH 6.5 and pH 7.4 medium, as well as normally cultured H cells, was determined by colony formation assay before and after treatment with 8 μM OXA. As shown in Figure 2B, H^R^ cells have stronger clonogenic ability than do H after being treated with 8 μM OXA for 24 h, especially at pH 6.5. The similar results were obtained in the B and B^R^ cells.

One of the main features of EMT is decreased expression of E‐cadherin, α‐catenin and β‐catenin as markers of epithelial cells and the increased expression of vimentin and fibronectin as markers of mesenchymal cells.^35^ Metalloproteinases (MMPs) (mainly MMP2 and MMP9) have a vital function in cell regeneration, programmed death, angiogenesis and many other tissue functions, and they participate in normal development and pathological processes, such as inhibition of NF‐kB, EMT and cellular invasion.^36^, ^37^ To detect the EMT of the HCC drug‐resistant cells at the molecular level and its correlation with the extracellular acidic environment, we determined the expression levels of EMT marker molecules by western blotting in drug‐resistant cells and sensitive cells. Compared with sensitive cells H, the levels of E‐cadherin, α‐catenin and β‐catenin were decreased, and the expression levels of vimentin, fibronectin, MMP2 and MMP9 were increased in drug‐resistant cells H^R^, especially at pH 6.5 (Figure 2C). Also, compared with values in B cells, E‐cadherin, α‐catenin and β‐catenin were significantly decreased, while values of vimentin, fibronectin, MMP2 and MMP9 were significantly increased in B^R^ cells, especially at pH 6.5 (Figure 2C).

An important mechanism for tumour resistance is that of EMT enhancing invasion and migration in cancer cells.^36^, ^37^ We found that, compared with counts in sensitive cells, the counts of the two drug‐resistant cell lines that passed through Transwell pores were significantly increased (Figure 2D) and had a larger scratch healing area (Figure 2E) after culture for 48 h, especially at pH 6.5.

Taken together, these data indicate that a significant EMT occurred in H^R^ cells and was positively correlated with the extracellular acid environment, which also means that it was correlated with the level of ASIC1a.

### 3.3 Inhibiting the activity of ASIC1a suppresses the EMT and enhances the chemosensitivity of H^R^ and B^R^ cells

The increase in ASIC1a expression (Figure 1) and the occurrence of EMT that correlated with ASIC1a expression (Figure 2) in H^R^ and B^R^ cells prompted us to diminish the amount of ASIC1a with PcTx1 (a potent and specific blocker of the ASIC1a) to elucidate the direct effect of ASIC1a on the chemosensitivity of H^R^ and B^R^ cells and the relationship with EMT.

**FIGURE 3:**
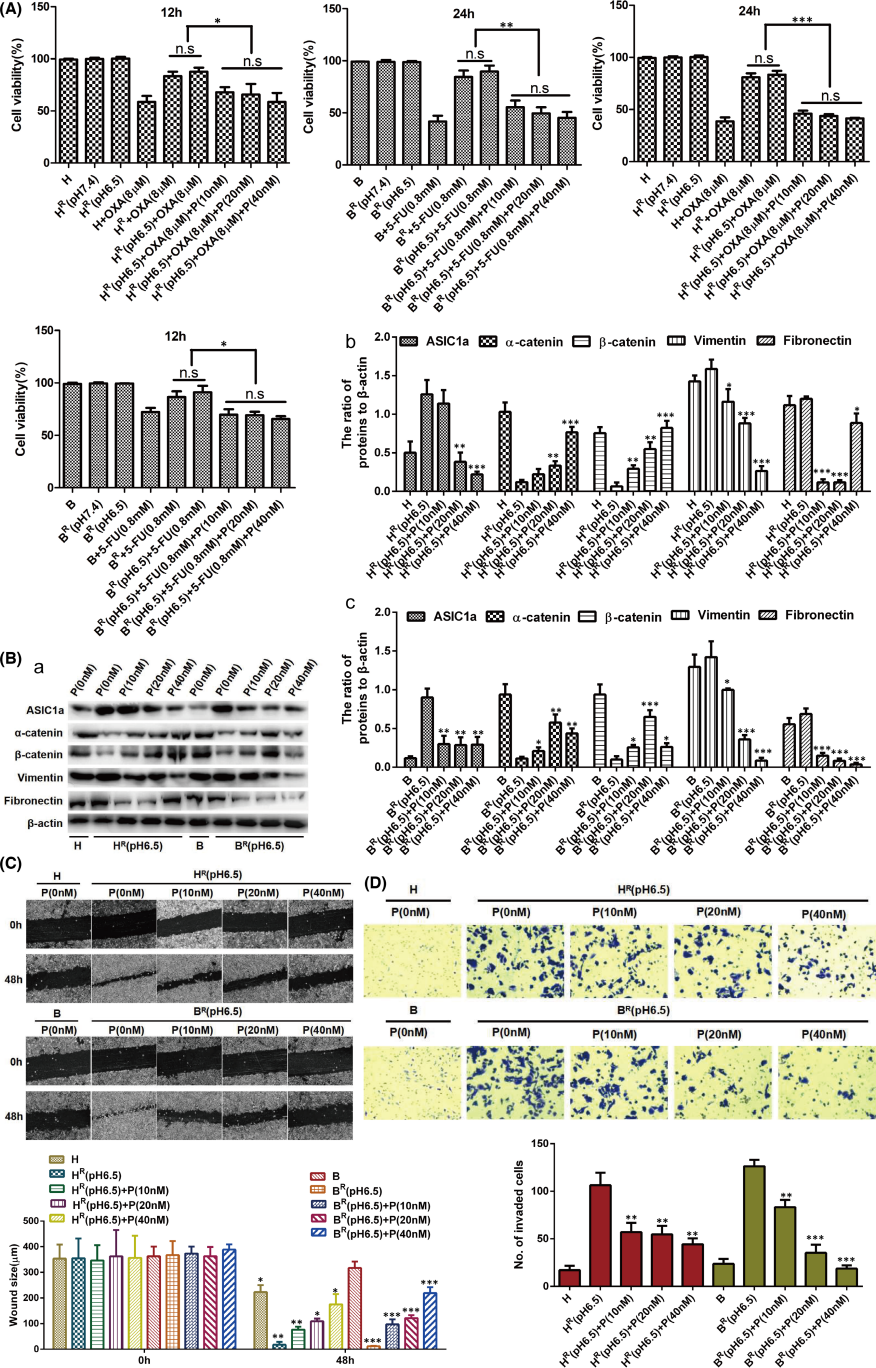
Inhibiting the activity of ASIC1a suppresses the EMT and enhances the chemosensitivity of H^R^ and B^R^ cells. (A) MTT analysis of the cell viability. Data were expressed as the mean ± SD, *n* = 3. ns, no significance, *p < 0.05, **p < 0.01, ***p < 0.001. (B) Western blotting analysis. Data were expressed as the mean ± SD, *n* = 3. *p < 0.05, **p < 0.01 and ***p < 0.001 all versus H^R^(pH6.5) or B^R^(pH 6.5) group. (C) Migration abilities of different group cells were examined by wound‐healing assays. Representative images were taken at ×100 magnification. Data were expressed as the mean ± SD, *n* = 3. *p < 0.05, **p < 0.01 and ***p < 0.01 all versus 0 h group. (D) Invasion abilities of different group cells were determined by transwell invasion assays. Representative images were taken at ×200 magnification. Data were expressed as the mean ± SD, *n* = 3. **p < 0.01 and ***p < 0.001 all versus H^R^(pH6.5) or B^R^(pH6.5) group

First, we detected the chemoresistance of H^R^ and B^R^ cells. The viability of H^R^ cells treated with 8 μM OXA and B^R^ cells treated with 0.8 mM 5‐FU and H^R^ and B^R^ cells treated with 10, 20 and 40 nM PcTx1 were assessed by MTT analysis at pH 6.5. As displayed in Figure 3A, compared with H^R^ cells treated with OXA and B^R^ cells treated with 5‐FU, H^R^ and B^R^ cells treated with PcTx1 were chemosensitive; no difference in cell viability of each group after treatment with PcTx1 was evident. We deduced that PcTx1 inhibited the level of ASIC1a, thereby reducing the chemical resistance of H^R^ and B^R^ cells in acidic medium (pH 6.5).

We next detected the expression levels of α‐catenin, β‐catenin, vimentin and fibronectin after treatment with 10, 20 and 40 nM PcTx1 at pH 6.5 by Western blotting in H^R^ and B^R^ cells. As shown in Figure 3Ba–c compared with untreated H^R^ and B^R^ cells in acidic medium, the expression of α‐catenin and β‐catenin was upregulated in H^R^ and B^R^ cells after treatment with 10, 20 and 40 nM PcTx1, whereas the levels of vimentin and fibronectin were decreased. These data suggest that inhibiting the activity of ASIC1a can inhibit EMT and, based on the statistical analysis (Figure 3Bb,c), and the principle of low cytotoxicity, 20 nM PcTx1, was selected as the drug concentration for further study.

Finally, at pH 6.5, we detected the levels of migration and invasion in H^R^ and B^R^ cells after treatment with 10, 20 and 40 nM PcTx1 by scratch healing assay and Transwell invasion assay. Compared with untreated H^R^ and B^R^ cells in acidic medium, the scratch area and number of invaded cells of H^R^ and B^R^ cells were significantly reduced after treatment with 10, 20 and 40 nM PcTx1 (Figure 3C,D). These data indicate that inhibiting the activity of ASIC1a reduced the migration and invasion in H^R^ and B^R^ cells.

### 3.4 ASIC1a knockdown suppresses the EMT and enhances the chemosensitivity of H^R^ and B^R^ cells

**FIGURE 4:**
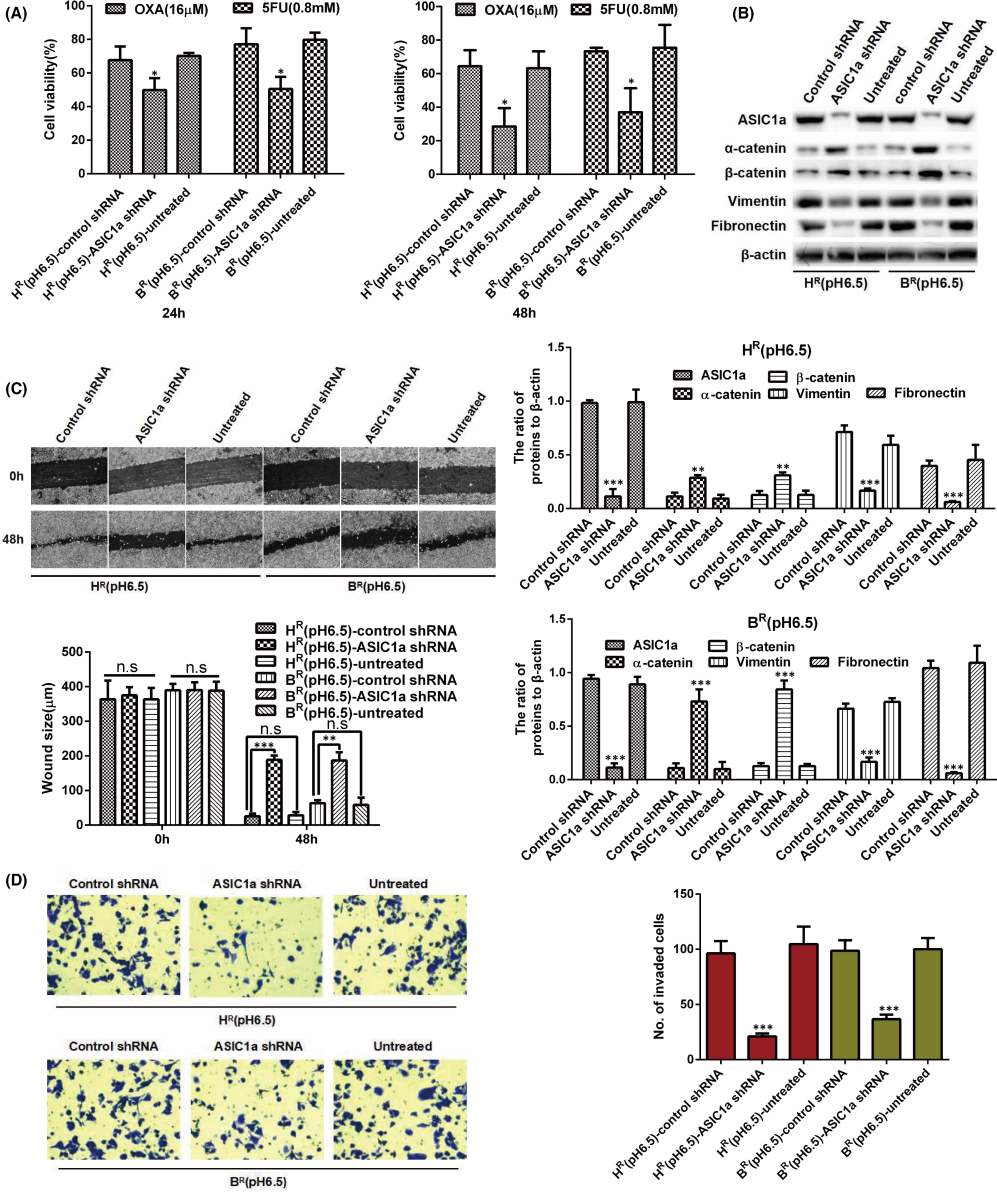
ASIC1a knockdown suppresses the EMT and enhances the chemosensitivity of H^R^ and B^R^ cells. (A) MTT analysis of the cell viability. Data were expressed as the mean ± SD, *n* = 3. *p < 0.05 versus H^R^(pH6.5)‐control shRNA or B^R^(pH 6.5)‐control shRNA group. (B) Western blotting analysis. Data were expressed as the mean ± SD, *n* = 3. **p < 0.01 and ***p < 0.001 all versus Control shRNA group. (C) Migration abilities of different group cells were examined by wound‐healing assays. Representative images were taken at ×100 magnification. Data were expressed as the mean ± SD, *n* = 3. ns, no significance, **p < 0.01, ***p < 0.001. (D) Invasion abilities of different group cells were determined by transwell invasion assays. Representative images were taken at ×200 magnification. Data were expressed as the mean ± SD, *n* = 3. ***p < 0.001 versus H^R^(pH6.5)‐control shRNA or B^R^(pH6.5)‐control shRNA group

After ASIC1a shRNA was transfected, the results of MTT evaluation revealed that the cytochemical sensitivity of H^R^ and B^R^ cells was enhanced (Figure 4A). In addition, as indicated in Figure 4B, the level of ASIC1a, vimentin and fibronectin was downregulated, whereas the level of α‐catenin and β‐catenin was significantly increased with ASIC1a knockdown. We then detected the migration and invasion of H^R^ and B^R^ cells after ASIC1a shRNA transfection by scratch healing assay and the Transwell invasion assay. In acidic medium, the scratch area and number of invaded cells of H^R^ and B^R^ cells were significantly reduced after the transfection (Figure 4C,D). These data indicate that silencing the ASIC1a gene could inhibit the migration and invasion of H^R^ and B^R^ cells.

### 3.5 Overexpression of ASIC1a gene promotes the EMT and reduces the chemosensitivity of H^R^ and B^R^ cells

**FIGURE 5:**
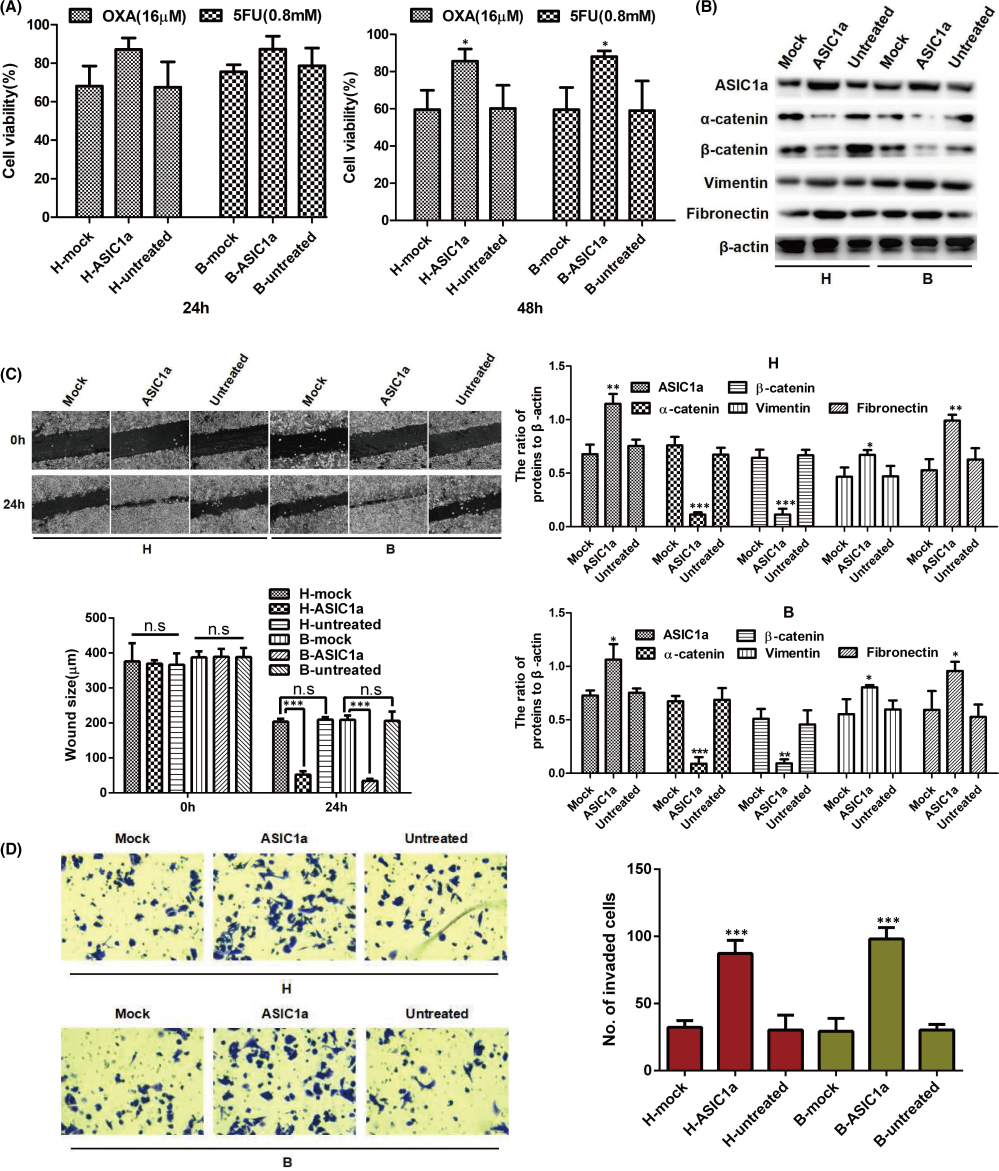
Overexpression of ASIC1a gene promotes the EMT and reduces the chemosensitivity of H^R^ and B^R^ cells. (A) MTT analysis of the cell viability. Data were expressed as the mean ± SD, *n* = 3. *p < 0.05 versus H^R^(pH6.5)‐mock or B^R^(pH6.5)‐mock group. (B) Western blotting analysis. Data were expressed as the mean ±SD, *n* = 3. *p < 0.05, **p < 0.01 and ***p < 0.001 all versus Mock group. (C) Migration abilities of different group cells were examined by wound‐healing assays. Representative images were taken at ×100 magnification. Data were expressed as the mean ± SD, *n* = 3. n.s: no significance, ***p < 0.001. (D) Invasion abilities of different group cells were determined by transwell invasion assays. Representative images were taken at ×200 magnification. Data were expressed as the mean ± SD, *n* = 3. ***p < 0.001 versus H^R^(pH6.5)‐mock or B^R^(pH6.5)‐mock group

After confirming the relationship among the suppression of ASIC1a and the chemosensitivity of H^R^ and B^R^ cells as well as EMT level in an acidic medium, we used ASIC1a overexpression in H and B cells to explore the chemosensitivity of H^R^ and B^R^ cells and its relationship with EMT in acidic medium. As displayed in Figure 5A, the cell viability, as measured with MTT assay, was increased in B and H transfectant cells. We then determined the expression of α‐catenin, β‐catenin, vimentin and fibronectin by Western blot. Overexpressed ASIC1a upregulated vimentin and fibronectin and downregulated α‐catenin and β‐catenin expression (Figure 5B). Next, we detected the levels of migration and invasion in H and B cells after transfection of SIC1a expression vector by scratch healing assay and the Transwell invasion assay. In acidic medium, the scratch area and number of invaded H and B cells were significantly increased after the transfection (Figure 5C,D). These data indicate that overexpressed ASIC1a gene can stimulate the migration and invasion of H and B cells.

### 3.6 ASIC1a mediates EMT in H^R^ and B^R^ cells

**FIGURE 6:**
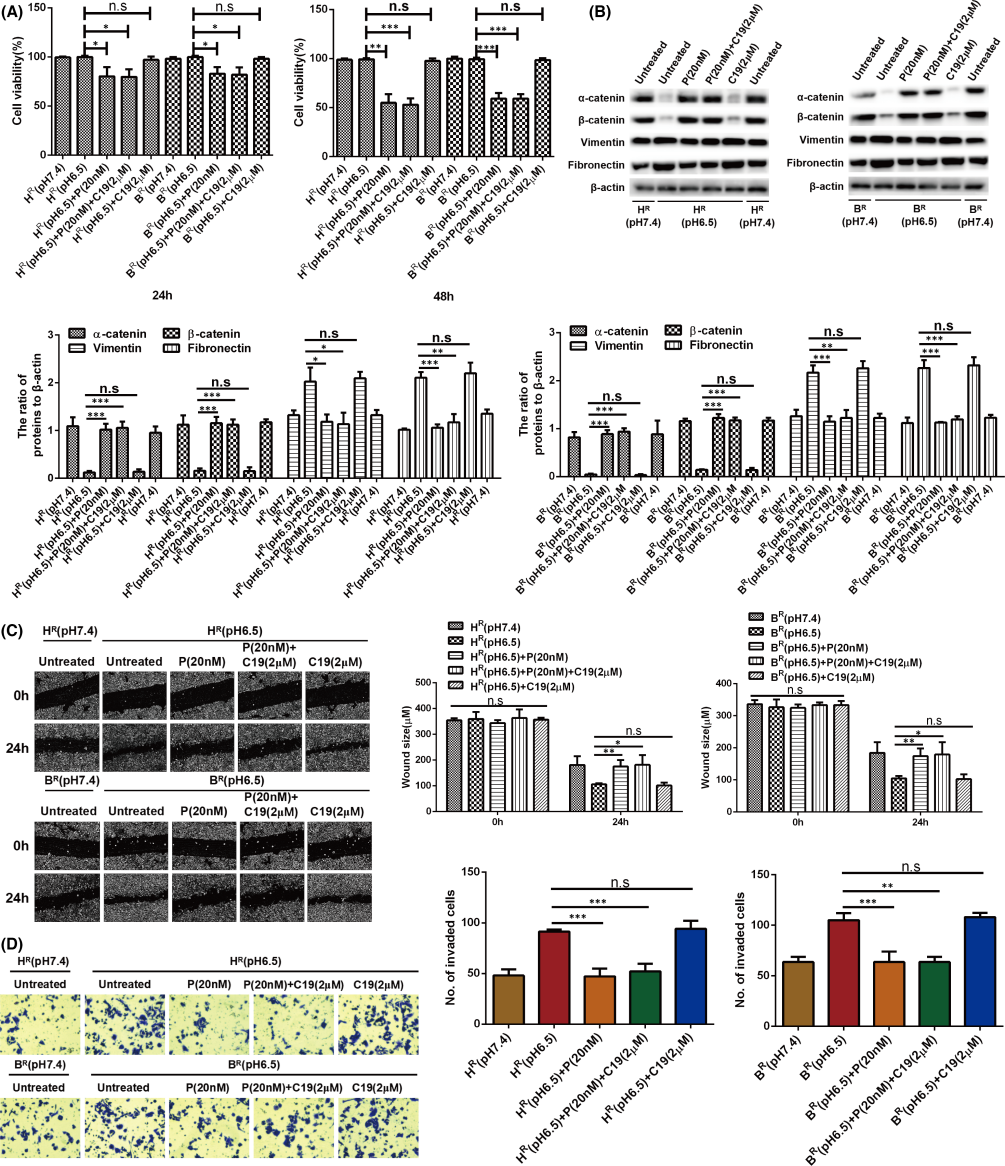
ASIC1a mediates EMT in H^R^ and B^R^ cells. (**A**) MTT analysis of the cell viability. Data were expressed as the mean ± SD, *n* = 3. ns, no significance, *p < 0.05, **p < 0.01, ***p < 0.001. (B) Western blotting analysis. Data were expressed as the mean ± SD, *n* = 3. ns, no significance, *p < 0.05, **p < 0.01, ***p < 0.001. (C) Migration abilities of different group cells were examined by wound‐healing assays. Representative images were taken at ×100 magnification. Data were expressed as the mean ± SD, *n* = 3. ns, no significance, *p < 0.05, **p < 0.01. (D) Invasion abilities of different group cells were determined by transwell invasion assays. Representative images were taken at ×200 magnification. Data were expressed as the mean ± SD, *n* = 3. ns, no significance, **p < 0.01, ***p < 0.001

As ASIC1a and EMT were increased in H^R^ and B^R^ cells and suppressed by inhibiting the activity of ASIC1a, we explored whether EMT was mediated via ASIC1a. As shown in Figure 6A, the acid‐induced cell viability of H^R^ and B^R^ cells was significantly attenuated by PcTx1. The acid‐induced cell viability of H^R^ and B^R^ cells was not decreased by C19 to block the occurrence of EMT. However, the acid‐induced cell viability of H^R^ and B^R^ cells was decreased in the presence of PcTx1 and C19.

We then measured the acid‐induced expression of α‐catenin, β‐catenin, vimentin and fibronectin. As displayed in Figure 6B, the acid‐induced low expression levels of α and β‐catenin were significantly increased, and the acid‐induced high expression levels of vimentin and fibronectin were reduced by PcTx1 in H^R^ and B^R^ cells. The acid‐induced low expression levels of α and β‐catenin were not increased, and the acid‐induced high expression levels of vimentin and fibronectin were not reduced, by C19 in H^R^ and B^R^ cells. However, the acid‐induced expression levels of α and β‐catenin, vimentin and fibronectin were changed by PcTx1 and C19 in the presence of PcTx1 in H^R^ and B^R^ cells. Next, the acid‐induced levels of migration and invasion in H^R^ and B^R^ cells were not decreased by C19, whereas they were decreased in the presence of PcTx1 and C19 (Figure 6C,D).

Overall, the EMT induced by the acidic environment was reduced by PcTx1 to reduce activation of ASIC1a. These data reveal that ASIC1a has a preeminent effect on mediating EMT in H^R^ and B^R^ cells.

### 3.7 ASIC1a reduces the chemosensitivity of H^R^ and B^R^ cells by promoting EMT via the AKT/GSK3β/Snail signalling pathway, which was driven by TGFβ/Smad signals

**FIGURE 7:**
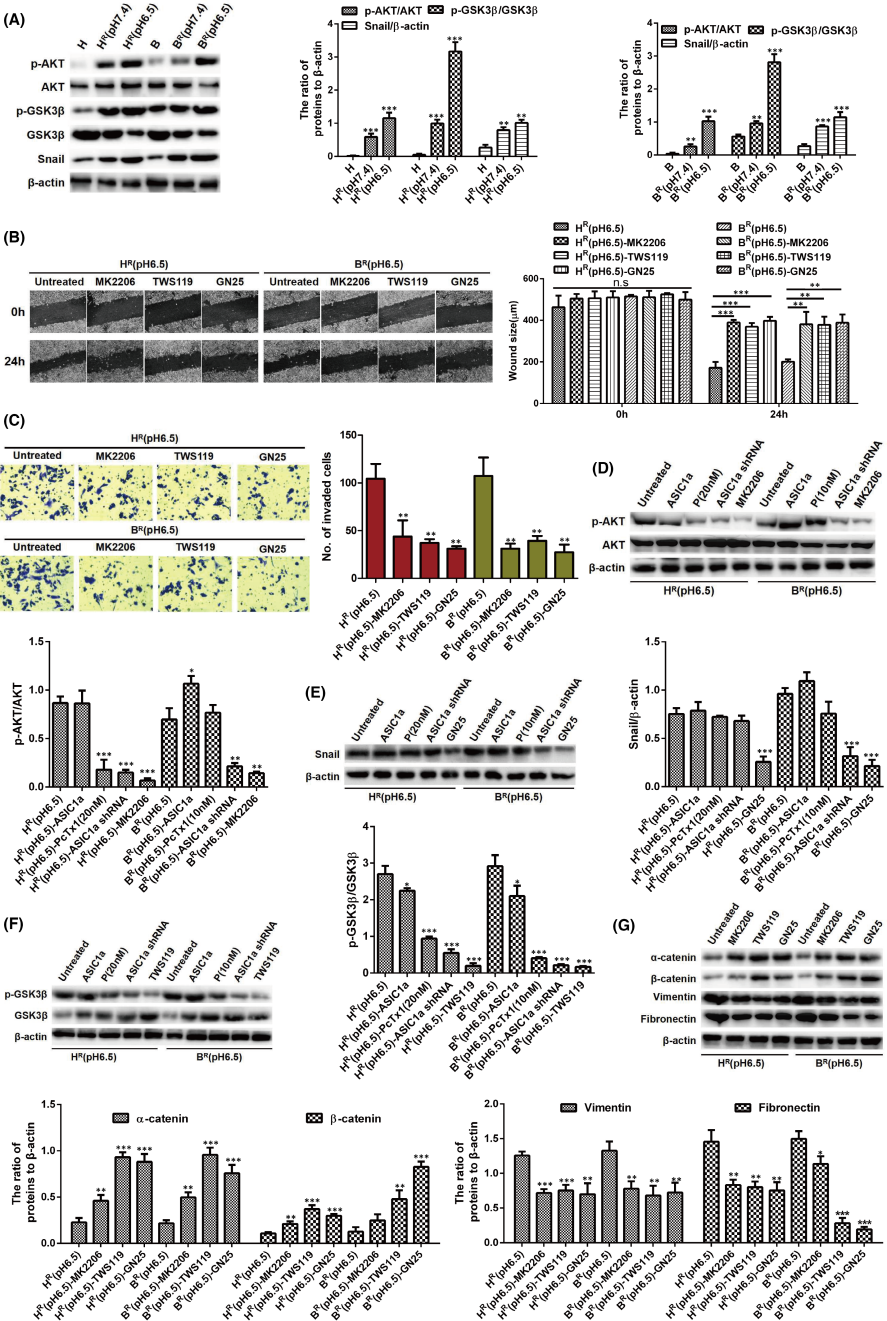
ASIC1a reduces the chemosensitivity of H^R^ and B^R^ cells by promoting EMT via the AKT/GSK3β/Snail signalling pathway which was driven by TGFβ/Smad signals. (A) Western blotting analysis. Data were expressed as the mean ± SD, *n* = 3. **p < 0.01 and ***p < 0.001 versus H or B group. (B) Migration abilities of different group cells were examined by wound‐healing assays. Representative images were taken at ×100 magnification. Data were expressed as the mean ± SD, *n* = 3. ns, no significance, **p < 0.01, ***p < 0.001. (C) Invasion abilities of different group cells were determined by transwell invasion assays. Representative images were taken at ×200 magnification. Data were expressed as the mean ± SD, *n* = 3. **p < 0.01 versus H^R^(pH6.5) or B^R^(pH6.5) group. (D–G) Western blotting analysis. Data were expressed as the mean ± SD, *n* = 3. *p < 0.05, **p < 0.01 and ***p < 0.001 versus H^R^(pH6.5) or B^R^(pH6.5) group

Early studies revealed that the AKT/GSK3β/Snail pathway touches upon the regulation of EMT,^38^, ^39^ but whether ASIC1a is involved has been unknown. We found, by Western blot analysis, that the levels of Snail and the p‐AKT and p‐GSK3β were upregulated in H^R^ (pH 6.5) and B^R^ (pH 6.5) cells compared with levels in H and B cells (Figure 7A).

At pH 6.5, we detected the levels of migration and invasion in H^R^ and B^R^ cells after treatment with MK2206, TWS119 and GN25 by scratch healing assay and Transwell invasion assay. Compared with untreated H^R^ and B^R^ cells in acidic medium, the scratch healing area and number of invaded cells of H^R^ and B^R^ cells were significantly reduced after treatment with MK2206, TWS119 and GN25 (Figure 7B,C).

Additionally, compared with protein expression levels in untreated H^R^ and B^R^ cells in acidic medium, the levels of Snail (Figure 7D) and the phosphorylation levels of AKT (Figure 7E) and GSK3β (Figure 7F) were significantly downregulated after treatment with PcTx1 or ASIC1a shRNA in H^R^ (pH 6.5) and B^R^ (pH 6.5) cells. We also evaluated the effect of the AKT/GSK3β/Snail pathway on EMT in H^R^ and B^R^ cells by Western blotting. As displayed in Figure 7G, compared with levels in untreated H^R^ and B^R^ cells in acidic medium, the levels of α‐catenin and β‐catenin were increased, whereas the levels of vimentin and fibronectin were reduced after treatment with MK2206, TWS119 and GN25 in H^R^ and B^R^ cells.

As AKT/GSK3β/Snail pathway is driven upstream by the cytokine TGFβ (one of the main inducers of EMT) via Smad signalling, we explored the relationship between the AKT/GSK3β/Snail pathway and TGFβ/Smad signalling in HCC cells. TGFβ/Smad signals were activated in resistant HCC cells at pH 6.5 (Figure S3A) and in ASIC1a‐overexpressed HCC cells (Figure S3B). Also, the AKT/GSK3β/Snail pathway was inhibited by LY2157299 (a TGFβ/Smad inhibitor) (Figure S3C) and activated by TGF‐β1 (10 ng/ml) (Figure S3D). These results indicate that AKT/GSK3β/Snail is driven by TGFβ/Smad signals in resistant HCC cells.

## 4 DISCUSSION

Due to their rapid growth, tumour cells often form an hypoxic environment.^40^ Hypoxia can transform cell metabolism and increase acidosis, thereby producing a high acid load.^41^ The tumour cells can adapt to the acidic microenvironment by turning on the detoxification mode.^42^ Extracellular acidification is an important feature of the cancer microenvironment, as it can control the migration and invasion of tumour cells by influencing immune cell function, clonal cell evolution and drug tolerance.^43^, ^44^, ^45^ As an acid sensor, ASIC1a promotes the development of malignant tumours by promoting the migration and invasion of tumour cells. In HCC cells, ASIC1a enhances drug tolerance by mediating Ca^2+^ influx.^20^ EMT is a necessary transformation process for the local and long‐distance progression of many malignant tumours, including HCC.^46^EMT has enhanced tumour cell invasion and metastasis for tumour resistance.^36^, ^37^ However, the interaction between ASIC1a and EMT in tumour resistance has not been defined. In this study, the identification of ASIC1a and EMT functions and their molecular mechanisms and interactions in resistant HCC cells provided a reasonable explanation for low pH being advantageous for the development of resistant cancer cells, and it provides new ideas for reversing tumour drug resistance, that is by inhibiting EMT.

Consistent with previous studies, the protein level of ASIC1a in HCC tissues and resistant cells was higher than that in parental cells, especially at pH 6.5.^20^ In addition, immunofluorescence and immunohistochemistry staining showed that membrane ASIC1a has a considerably higher expression in H^R^ and B^R^ cells at pH 6.5. Quantitative real‐time PCR analysis revealed that the mRNA level of ASIC1a in HCC tissues was higher than in adjacent non‐tumour tissues, and the mRNA of ASIC1a was expressed more in HCC‐resistant cells than in sensitive cells. We also found a morphological cellular change, that is mesenchymal phenotype, that is occurred in resistant HCC cells, especially at pH 6.5. These data are evidence that ASIC1a stimulates the development of malignant and resistant tumour cells at low pH.

EMT had been thought involved only in the invasion and metastasis of malignant cells. In recent years, though, it has been learned that EMT occurring in cancer cells could also inhibit drug resistance and apoptosis, which are indispensable components of tumour resistance.^36^, ^37^ Thus, reversing EMT or killing cancer cells with the EMT phenotype has been proposed as a potential treatment for cancers. This study detected EMT through characterization of cell proliferation, clone formation, migration, invasion and molecular marker expression in vitro. In previous studies, as a strong inducer of EMT, transforming growth factor‐β has carcinogenic effects, showing that the level of cytokines in advanced HCC is elevated, which also makes epithelial plasticity a hot issue in HCC.^47^ In this study, we found significant EMT in HCC‐resistant cells, especially at pH 6.5. This observation suggests that acidic extracellular pH promotes EMT, and up‐regulation of ASIC1a may promote EMT in HCC. However, the relationship between ASIC1a and EMT and their relationship to drug resistance is not well understood. Therefore, we inhibited the expression of ASIC1a through PcTx1 to determine the role of ASIC1a in resistance and EMT of H^R^ and B^R^ cells by measuring cell proliferation, clone formation, migration, invasion and molecular marker expression *in vitro*. Results of these studies revealed that at pH 6.5, inactivation of ASIC1a by PcTx1 could enhance the chemosensitivity of H^R^ and B^R^ cells and partially inhibit EMT and promote its reversal, mesenchymal to epithelial transition (MET). In addition, ASIC1a gene knockdown heightened the chemosensitivity of H^R^ and B^R^ cells and inhibited EMT in those cells. From the above results, we deduced that ASIC1a may be liable for EMT and altered drug‐resistant in HCC cells.

The preliminary results of this study showed that the expression level of E‐cadherin in B, B^R^ (pH 7.4) and B^R^ (pH 6.5) cells was low without difference among the cells, while levels of other epithelial markers (α‐catenin and β‐catenin) and mesenchyme markers (vimentin and fibronectin) had significant differences between sensitive and resistant cells of HepG2 and Bel7402, especially at pH 6.5, as shown in Figure 2C. These results indicate that E‐cadherin may not be the main marker of epithelial–mesenchymal transition mediated by an acidic environment in drug‐resistant cells, especially in Bel7042. To extensively study the influence of an acidic environment on the occurrence of EMT and drug resistance of tumour cells, we only focused on detecting the expression levels of epithelial markers (α‐catenin and β‐catenin) and mesenchymal markers (vimentin and fibronectin) in follow‐up studies (Figures 3, 4, 5, 6, 7). Based on the above results, we only showed the expression of E‐cadherin in Figure 2, but not in other blots.

Part of the activity of EMT in tumour cells is believed to heighten the cells’ invasiveness, generate circulating cancer cells and cancer stem cells, and enhance their resistance to anticancer drugs.^48^ Several transcriptional repressors, including Snail (SNAI1), Slug (SNAI2) and ZEB family, play a key role of EMT in cancer and development.^28^, ^29^ Regulating Akt/GSK3β/Snail signal transduction axis can inhibit tumour cell EMT and chemoresistance.^26^, ^27^ Extracellular acidosis leads to drug tolerance, including decreased apoptotic latent stimulation of autophagy, suppression of immunity and genetic changes.^49^ It has been reported that ASIC1a mediates tumour tolerance by inducing calcium influx and preventing apoptosis through induction of autophagy.^20^, ^50^ Also, extracellular acidification activates ASIC1a to facilitate cancer cell migration and adhesion.^34^ Based on this knowledge, we questioned whether ASIC1a‐mediated EMT induces tumour tolerance through the AKT/GSK3β/Snail pathway. Accordingly, we documented that the AKT/GSK3β/Snail activity of drug‐resistant cell lines H^R^ and B^R^ was higher than that of sensitive cells. Inhibition of the AKT/GSK3β/Snail pathway with MK2206, TWS119 or GN25 reversed the EMT of H^R^ and B^R^ cells. As the AKT/GSK3β/Snail pathway is driven upstream by cytokine TGFβ (one of the main inducers of EMT) via Smad signalling, we explored the relationship between the AKT/GSK3β/Snail pathway and TGFβ/Smad signalling in HCC cells. TGFβ/Smad signals were activated in resistant HCC cells at pH 6.5 and in ASIC1a‐overexpressed HCC cells. Also, the AKT/GSK3β/Snail pathway was inhibited by LY2157299 (a TGFβ/Smad inhibitor) (5 μM) and activated by TGF‐β1 (10 ng/ml). In all, the above results indicate that AKT/GSK3β/Snail is driven by TGFβ/Smad signals in resistant HCC cells. However, whether AKT/GSK3β/Snail‐mediated EMT is driven by TGFβ/Smad signals is not known and will be examined in our follow‐up study.

## 5 CONCLUSIONS

**FIGURE 8:**
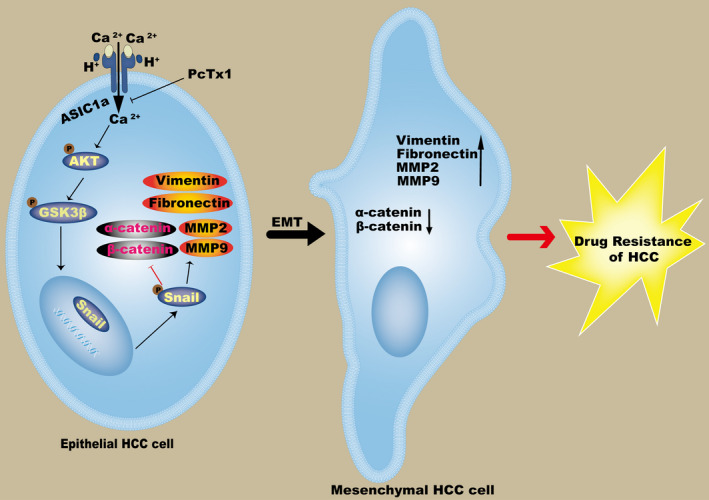
Schematic diagram illustrates that ASIC1a mediates the drug resistance of HCC by promoting EMT via the AKT/GSK3β/Snail signalling pathway

Activation of ASIC1a by extracellular acidification through the AKT/GSK3β/Snail pathway stimulates EMT to help make HCC chemotherapy resistant (Figure 8). This finding suggests that ASIC1a can reverse EMT, prevent HCC cells from developing drug resistance and control the progression of HCC. However, this study did not explore the relationship between calcium influx and EMT. Therefore, other potential mechanisms of ASIC1a and EMT participation in the regulation of drug resistance in HCC cells remain to be studied.

## CONFLICT OF INTEREST

The authors declare that they have no competing interests.

## AUTHOR CONTRIBUTIONS

**Yinci Zhang:** Conceptualization (lead); Data curation (lead); Formal analysis (lead); Investigation (lead); Methodology (lead); Writing – original draft (lead); Writing – review & editing (lead). **Niandie Cao:** Conceptualization (equal); Data curation (equal); Formal analysis (equal); Investigation (equal); Methodology (equal); Project administration (equal); Supervision (equal); Validation (equal). **Jiafeng Gao:** Conceptualization (equal); Data curation (equal); Formal analysis (equal); Investigation (equal); Methodology (equal); Project administration (equal); Software (equal); Supervision (equal); Validation (equal). **Jiaojiao Liang:** Conceptualization (equal); Data curation (equal); Formal analysis (equal); Investigation (equal); Methodology (equal); Resources (equal); Software (equal); Supervision (equal); Validation (equal); Visualization (equal). **Yong Liang:** Conceptualization (supporting); Data curation (supporting); Formal analysis (supporting); Investigation (supporting); Methodology (supporting); Project administration (supporting); Resources (supporting); Software (supporting); Supervision (equal); Validation (equal); Visualization (equal). **Yinghai Xie:** Conceptualization (supporting); Data curation (supporting); Formal analysis (supporting); Investigation (supporting); Methodology (supporting); Project administration (supporting); Resources (supporting); Software (supporting); Supervision (equal); Validation (equal); Visualization (equal). **Shuping Zhou:** Conceptualization (supporting); Data curation (supporting); Formal analysis (supporting); Funding acquisition (supporting); Investigation (supporting); Methodology (supporting); Project administration (supporting); Resources (supporting). **Xiaolong Tang:** Conceptualization (equal); Data curation (equal); Formal analysis (equal); Funding acquisition (lead); Investigation (equal); Methodology (equal); Project administration (lead); Resources (lead); Software (equal); Supervision (lead); Validation (lead); Writing – original draft (equal); Writing – review & editing (lead).

## CONSENT FOR PUBLICATION

All authors are in agreement with the content of the manuscript.

## Supporting information

Figure S1

Figure S2

Figure S3

## Data Availability

The data that support the findings of this study are available from the corresponding author upon reasonable request.
