# Supplementary figures and images for: ASIC1a stimulates the resistance of human hepatocellular carcinoma by promoting EMT via the AKT/GSK3β/Snail pathway driven by TGFβ/Smad signals
Source: J Cell Mol Med. 2022 Apr 14;26(10):2777–92. doi: 10.1111/jcmm.17288 (PMC9097844; doi:10.1111/jcmm.17288)

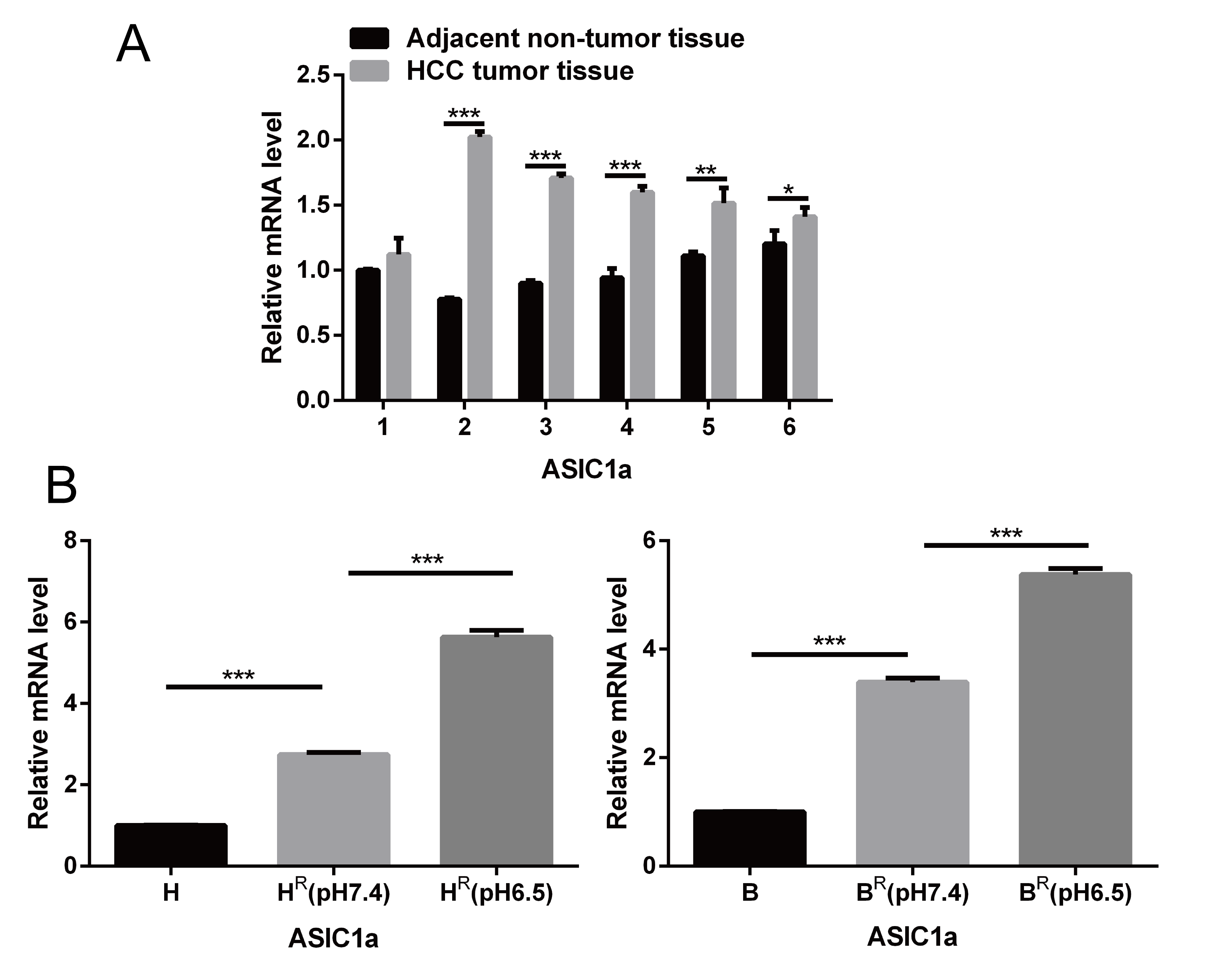

Supplement: Supplementary file 1 — Figure S1 [file JCMM-26-2777-s003.tif]

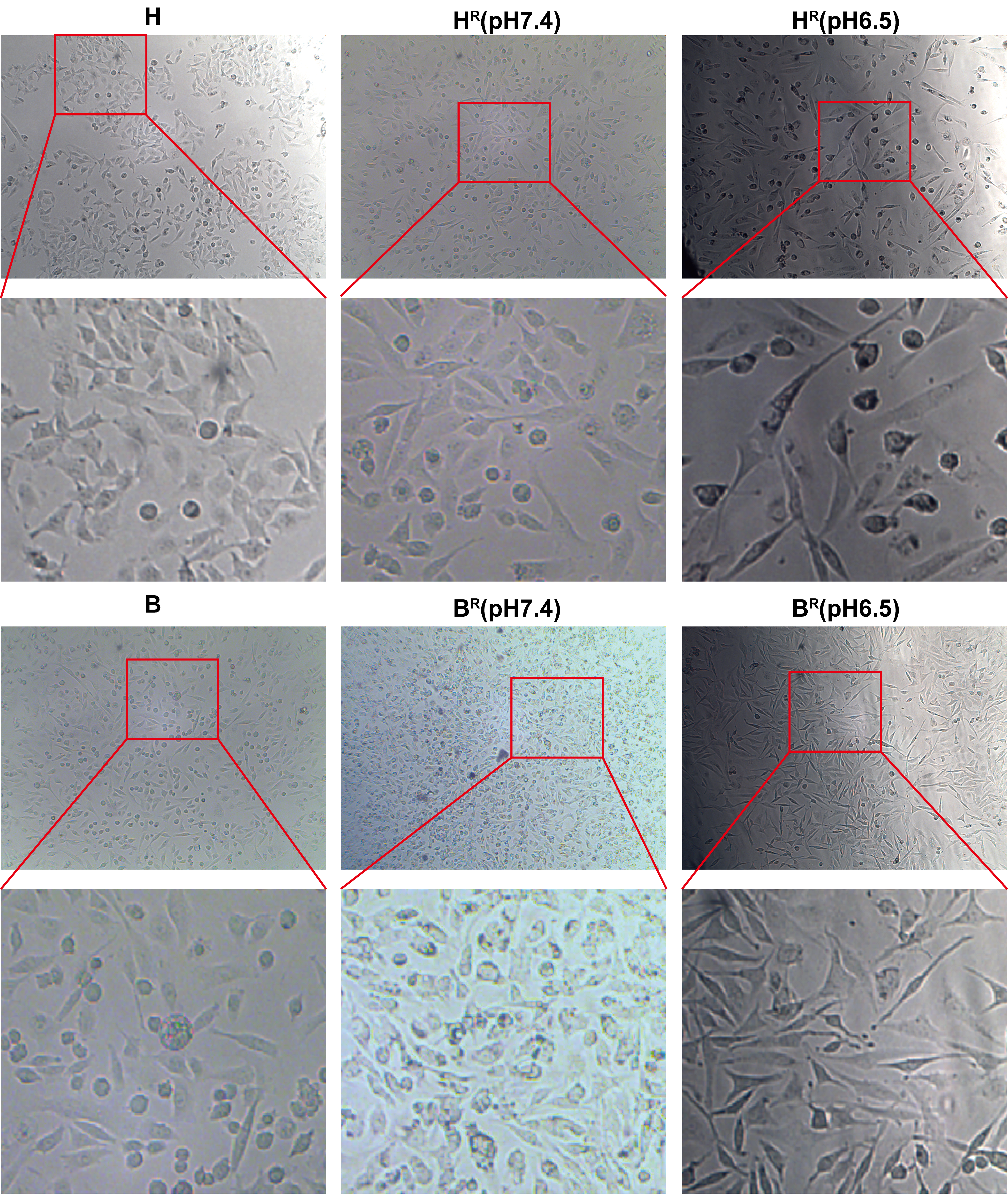

Supplement: Supplementary file 2 — Figure S2 [file JCMM-26-2777-s001.tif]

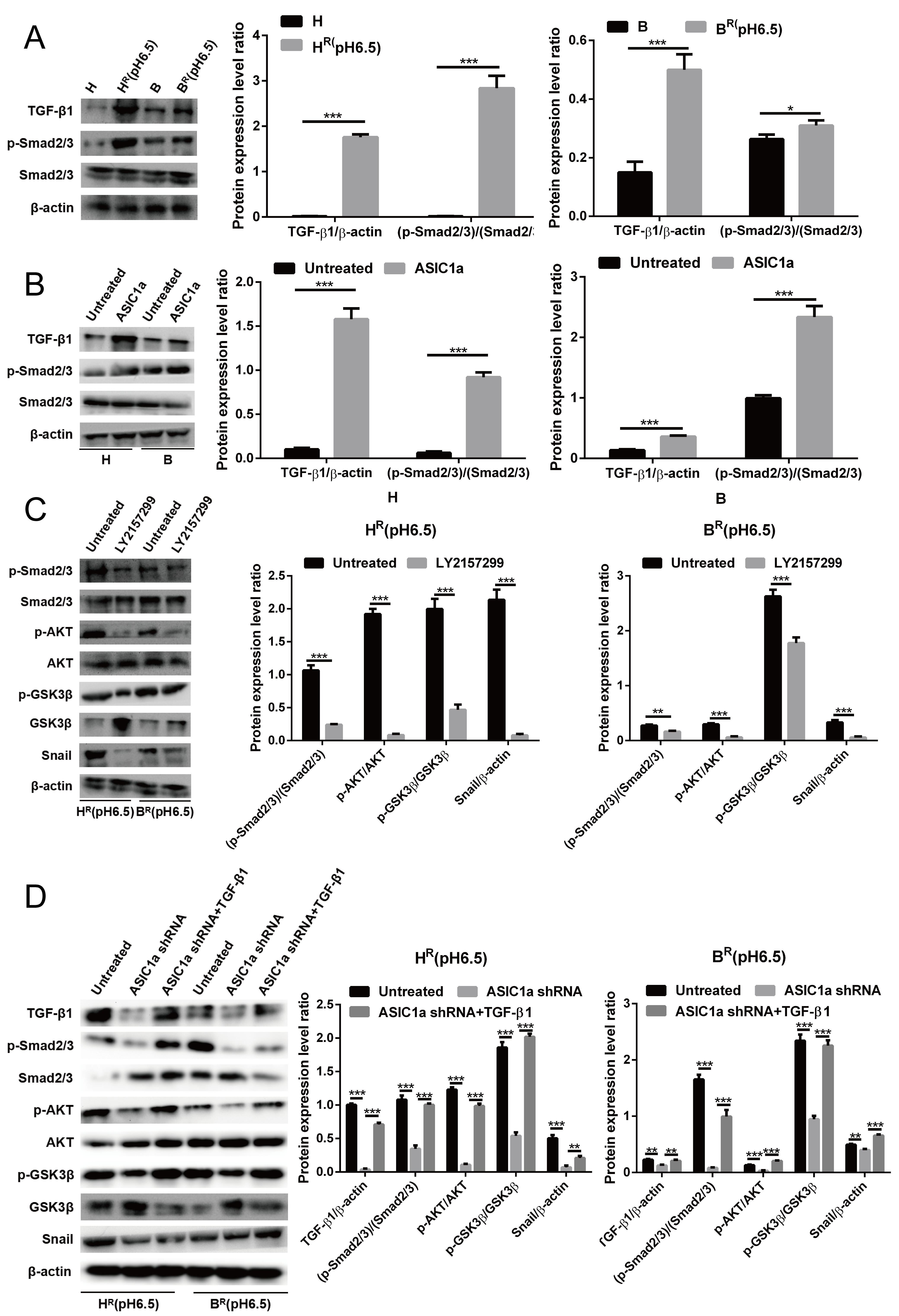

Supplement: Supplementary file 3 — Figure S3 [file JCMM-26-2777-s002.tif]
